# ^18^F-FDG-PET/CT to Detect Pathological Complete Response After Neoadjuvant Treatment in Patients with Cancer of the Esophagus or Gastroesophageal Junction: Accuracy and Long-Term Implications

**DOI:** 10.1007/s12029-023-00951-2

**Published:** 2023-07-01

**Authors:** D. C. van der Aa, S. S. Gisbertz, M. C. J. Anderegg, S. M. Lagarde, R. Klaassen, S. L. Meijer, S. van Dieren, MCCM Hulshof, JJGHM Bergman, R. J. Bennink, H. W. M. van Laarhoven, M. I. van Berge Henegouwen

**Affiliations:** 1 Department of Surgery, Amsterdam UMC, location University of Amsterdam, Amsterdam, Netherlands; 2 Cancer Treatment and Quality of Life, Cancer Center Amsterdam, Amsterdam, Netherlands; 3 Amsterdam Gastroenterology Endocrinology Metabolism, Amsterdam, Netherlands; 4 Department of Surgery, Erasmus Medical Center, Rotterdam, Netherlands; 5 Department of Medical Oncology, Amsterdam UMC, location University of Amsterdam, Amsterdam, Netherlands; 6 Department of Pathology, Amsterdam UMC, location University of Amsterdam, Amsterdam, Netherlands; 7 Department of Radiotherapy, Amsterdam UMC, location University of Amsterdam, Amsterdam, Netherlands; 8 Department of Gastroenterology, Amsterdam UMC, location University of Amsterdam, Amsterdam, Netherlands; 9 Department of Radiology and Nuclear Medicine, Amsterdam UMC, location University of Amsterdam, Amsterdam, Netherlands

**Keywords:** Esophageal cancer, Complete pathologic response, Prediction

## Abstract

**Purpose:**

The curative strategy for patients with esophageal cancer without distant metastases consists of esophagectomy with preceding chemo(radio)therapy (CRT). In 10–40% of patients treated with CRT, no viable tumor is detectable in the resection specimen (pathological complete response (pCR)). This study aims to define the clinical outcomes of patients with a pCR and to assess the accuracy of post-CRT FDG-PET/CT in the detection of a pCR.

**Methods:**

Four hundred sixty-three patients with cancer of the esophagus or gastroesophageal junction who underwent esophageal resection after CRT between 1994 and 2013 were included. Patients were categorized as pathological complete responders or noncomplete responders. Standardized uptake value (SUV) ratios of 135 post-CRT FDG-PET/CTs were calculated and compared with the pathological findings in the corresponding resection specimens.

**Results:**

Of the 463 included patients, 85 (18.4%) patients had a pCR. During follow-up, 25 (29.4%) of these 85 patients developed recurrent disease. Both 5-year disease-free survival (5y-DFS) and 5-year overall survival (5y-OS) were significantly higher in complete responders compared to noncomplete responders (5y-DFS 69.6% vs. 44.2%; *P* = 0.001 and 5y-OS 66.5% vs. 43.7%; *P* = 0.001). Not pCR, but only pN0 was identified as an independent predictor of (disease-free) survival.

**Conclusion:**

Patients with a pCR have a higher probability of survival compared to noncomplete responders. One third of patients with a pCR do develop recurrent disease, and pCR can therefore not be equated with cure. FDG**-**PET/CT was inaccurate to predict pCR and therefore cannot be used as a sole diagnostic tool to predict pCR after CRT for esophageal cancer.

## Introduction

Esophageal cancer has a dismal prognosis, due to its rapid dissemination regionally as well as to distant sites, leading to poor overall survival rates [1, 2]. The preferred treatment strategy for patients without distant metastases consists of esophagectomy with preceding chemoradiotherapy [3] or chemotherapy [4]. In some patients, neoadjuvant treatment is so effective that after surgery, no viable tumor cells are found neither in the esophagus nor in the resected lymph nodes. This phenomenon is called a pathological complete response (pCR) and is observed in 10–40% of esophageal cancer patients [5]. In general, it can be stated that a pCR is achieved more frequently in squamous cell carcinoma compared to adenocarcinoma and after neoadjuvant chemoradiotherapy compared to chemotherapy [6, 7]. Several studies have described a profound survival benefit after a pCR compared to scenarios of no/partial response [8–11]. A large multicenter study in which 299 patients with a pCR were included showed that patients with a complete response to neoadjuvant therapy have a fairly good prospect of survival with a disease-specific 5-year survival rate up to 68% [12]. It is, however, noticeable that despite the fact that patients with pCR have signs of complete tumor eradication, disease recurrence rates of up to 40% are still reported in literature [8, 9, 12, 13]. In the above-mentioned multicenter study by Vallböhmer et al., a recurrence rate of 23.4% was observed among patients with a pCR [12]. A large majority of these recurrences (86%) consisted of distant metastases suggesting a suboptimal systemic treatment effect.

Since disease-specific survival in patients with a pCR is mainly determined by the presence or absence of distant metastases the need for an esophagectomy after a complete response on chemo(radio)therapy is being disputed. In order to thoroughly investigate the safety of omitting esophagectomy after a putative pCR, adequate identification of patients with a pCR is essential. Possible diagnostic instruments to evaluate response are endoscopy with biopsies, endoscopic ultrasound (EUS), computed tomography (CT), and Fluor-18 fluoro-2-deoxy-D-glucose positron emission tomography ([F18]FDG-PET)/CT. A combination of EUS and FDG-PET/CT has proven to be adequate in the detection of nodal and distant metastasis with a reported sensitivity between 83 and 94% [14, 15]. However, ruling out the presence of vital disease at the original tumor site has turned out to be an ongoing clinical challenge. In their recent meta-analysis, Cong et al. concluded that FDG-PET alone is an insufficient tool for the assessment of the pathological response of the primary tumor after chemoradiotherapy with a pooled sensitivity and specificity of 67% and 69%, respectively [16]. In the twelve studies included in this meta-analysis, the standardized uptake value (SUV) at the primary tumor site was used as the determinant of residual disease. A complicating factor in this context is the fact that SUV is not only determined by vital tumor cells but also by interfering effects of treatment, such as radiation induced esophagitis [17]. We therefore hypothesized that in order to be a reliable determinant, this SUV needs to be corrected for the metabolic effects of chemoradiotherapy on the esophagus itself.

The aim of this study was to assess the outcome, in terms of survival and disease recurrence rates, of patients who obtain a pCR compared with patients without a pCR after neoadjuvant treatment followed by surgery for esophageal cancer. Furthermore, we evaluated the accuracy of FDG-PET/CT in the preoperative identification of patients with a complete response of the primary tumor by using a new protocol with an adjusted SUV.

## Patients and Methods

### Patient Population

Between March 1994 and September 2013, all consecutive patients with histologically confirmed, not metastatic (cT1N + M0 or cT2-4aN0-3M0) squamous cell carcinoma, adenocarcinoma, or large-cell undifferentiated carcinoma of the esophagus or gastroesophageal junction (Siewert type II) who underwent esophageal resection after neoadjuvant chemo(radio)therapy were included in the present study. Patients were selected from a prospectively collected database at the Department of Surgery, Academic Medical Center at the University of Amsterdam, the Netherlands. Patients were not asked to provide informed consent for this specific study because the data were primarily recorded as part of standard care. The local ethics committee approved this approach and waived formal evaluation.

### Pretreatment Staging and Treatment Indication

Initial staging consisted of endoscopy with biopsy, endoscopic ultrasonography, external ultrasonography of the neck, and a cervicothoracoabdominal CT scan. A FDG-PET/CT scan was not part of the initial staging but was in some cases performed prior to referral.

### Neoadjuvant Therapy

Four neoadjuvant chemoradiotherapy regimens were employed in the present study. All patients received 23 fractions of 1.8 Gy (41.4 Gy) external-beam radiotherapy combined with weekly administered carboplatin (AUC2) and paclitaxel (50 mg/m^2^). In some patients, chemoradiation was combined with deep loco-regional hyperthermia as part of a study [18]. Additionally, as part of a phase II clinical trial in our center, a proportion of patients received panitumumab (human monoclonal antibody to the epidermal growth factor receptor), at a dose of 6 mg/kg in addition to the standard neoadjuvant chemoradiation [19].

Neoadjuvant chemotherapy was chosen when the majority of the tumor was located in the cardia. Patients were restaged after neoadjuvant chemo(radio)therapy with CT or FDG-PET/CT. Patients who developed distant metastasis during neoadjuvant treatment were excluded.

### FDG-PET/CT Imaging

Starting in 2011, all patients underwent a restaging FDG-PET/CT in the third week after completion of neoadjuvant chemoradiotherapy. The FDG-PET/CT was performed using a Philips Gemini TF-16 PET/CT scanner (Philips Medical Systems, Eindhoven, the Netherlands) with spatial resolution near the field of view center of 4.8 mm in transverse and axial directions. A CT scan in the supine position was acquired from the base of the skull to mid-thighs. The 12-channel helical CT scanning parameters were 120 kVp, 50 mA/slice, rotation time 0.75 s, and slice thickness/interval 3.0 mm. Both oral and intravenous (porto-venous phase) contrast was used. At 60 min after intravenous injection of 180–240 MBq of 18F-FDG, emission scans were acquired from the base of the skull to mid-thighs over 10 bed positions at 2 min per position. Image reconstruction employed a list-mode version of a maximum likelihood expectation maximization algorithm with a time-of-flight kernel applied in both the forward and back-projection operations. Quantitative analysis was performed using standardized uptake values (SUVs) and calculated as the maximum value 1 h after injection. CT data were used for attenuation correction. Images were viewed using Hermes Hybrid viewer software (Hermes Medical Solutions, Stockholm, Sweden).

In order to exclude the effect of radiation induced esophagitis, a ratio was used to evaluate the true uptake of the FDG tracer at the primary tumor site. The maximum SUV of the tumor and the mean and maximum SUV of nonaffected esophageal tissue at the most proximal end of the radiation field were determined. The SUV ratio was calculated by dividing the maximum SUV of the tumor by both the mean SUV and the maximum SUV of the nonaffected esophagus. The above-described SUV scoring was performed without knowledge of the eventual pathological outcomes.

### Surgery

During the inclusion period, different types of open and minimally invasive transthoracic and transhiatal surgery were performed, as previously described [20, 21]. Surgery was performed within 6–10 weeks after completion of neoadjuvant chemo(radio)therapy.

Between 1994 and 2000, patients underwent an open transthoracic or transhiatal esophagectomy. Patients with a tumor distal of the carina were enrolled in a randomized, controlled trial comparing the transhiatal and transthoracic procedure [21]. Based on the results of this trial, patients with a true esophageal tumor generally underwent a transthoracic resection in the period after 2000. Patients with a tumor located at the gastroesophageal junction who had a reduced performance status (unable to undergo transthoracic esophagectomy) underwent transhiatal esophagectomy. Between 2009 and 2011, a minimal invasive transthoracic procedure was performed as part of a randomized controlled trial [20] and based on the results of this trial, patients generally underwent minimally invasive transthoracic resection in the period after 2011.

Patients with unresectable tumors during exploratory surgery or macroscopically irradical resections (R2) and those who died in hospital after surgery were not included in the present study.

### Pathology

Pathological findings were described in a standardized format by an experienced gastrointestinal pathologist according to local and national protocols. The ypTNM-stage, differentiation grade, radicality, total number of resected lymph nodes, and total number of positive lymph nodes, including their location, were recorded. All lymph nodes were embedded completely for pathology evaluation. In the absence of macroscopic recognizable tumor, the complete circumference of the tubular esophagus at the site of the original tumor was inspected, and any abnormal-appearing tissue was paraffin-embedded in order to make an adequate assessment for the presence of residual tumor and the effects of therapy.

When no residual tumor cells were seen in the proximal, distal, and circumferential resection margins, the resection was classified as R0. If a vital tumor was microscopically present at the proximal, distal, or circumferential resection margin, it was considered to be microscopically positive (R1). To grade the response to therapy, the degree of histomorphologic regression was classified with use of the Mandard score [22]. Routine H&E staining was performed using a standardized protocol. On indication, keratin immuno-histochemical staining techniques were used as an aid to detect vital tumor cells or micrometastases.

Patients were classified as having a pathological complete response (pCR) if no vital tumor cells were detected in the esophagus (ypT0) nor in the resected lymph nodes (ypN0), while there were no clinical/intraoperative signs of distant metastases. Patients with a partial response or no response at all were classified as noncomplete responders.

### Follow-up

All patients were seen at the outpatient clinic at 3-month intervals during the first year and 6-month intervals during the second, third, and fourth year and once in the fifth year. After 5 years, follow-up was obtained by telephone from the patient or the patient’s family practitioner. Follow-up was extended to March 2014 ensuring a minimal potential follow-up of 4 months. Recurrence of disease was diagnosed on clinical grounds. When recurrence was suspected, additional investigations (CT, FDG-PET/CT, MRI, ultrasound) were performed.

### Statistics

Statistical calculations were performed by SPSS software, version 20.0 (SPSS, Chicago, IL).

Normality of data distribution was checked by visually inspecting the histograms and boxplots. Differences between groups for continuous data were tested by the Mann–Whitney *U* test or Students *t*-test according to the distribution of the data. To compare categorical data, the chi-square or Fisher exact test was used. The Mann–Whitney *U*-test was used to compare continuous variables. Multivariate Cox regression analysis was carried out to identify independent prognostic factors. All factors from the univariate analysis with a *P*-value less than 0.05 were entered in this multivariate analysis. *P*-values less than 0.05 (two-sided) were considered statistically significant.

In order to assess the diagnostic accuracy of FDG-PET/CT in the detection of patients with a complete response, the above-mentioned SUV ratios were compared with the eventual pathological findings in the resection specimen. The diagnostic value of SUV ratio was assessed over a range of cut-off values by calculating the positive predictive value, negative predictive value, sensitivity, and specificity.

## Results

### Patient Demographics and Clinical Characteristics

Between March 1994 and September 2013, 504 patients with cancer of the esophagus or gastroesophageal junction underwent esophageal resection after neoadjuvant chemo(radio)therapy. In 6 (1.2%) patients, distant metastases were detected intra-operatively. Fourteen (2.7%) patients underwent a salvage resection because of recurrent/residual disease after definitive chemoradiotherapy. Both groups were excluded. Twenty-one (4.2%) of the remaining 484 patients died due to postoperative complications. These patients were excluded as well.

Among the 463 included patients, 85 (18.4%) obtained a pCR on neoadjuvant treatment. Eight out of 88 patients treated with chemotherapy had a pCR (9.0%). In the 375 patients who underwent chemoradiotherapy, a pCR was found in 77 cases (20.5%).

Table 1 summarizes the baseline characteristics of both complete responders and noncomplete responders. Complete responders had a similar distribution of age and comorbidity compared to noncomplete responders, but a larger portion of them consisted of female patients (37.6% vs. 22.5%; *P* = 0.004). Significant differences were observed with respect to tumor histology and tumor location with higher representation of squamous cell carcinomas and mid-esophageal tumors in the group of complete responders. Groups did not differ in terms of clinical tumor — nor clinical nodal stage. In Table 2, neoadjuvant and surgical treatment is specified. A pCR was more frequently achieved after neoadjuvant chemoradiotherapy compared to neoadjuvant chemotherapy (*P* = 0.013). Additionally, a larger portion of complete responders had undergone a transthoracic resection compared to the noncomplete responders (84.7% vs. 67.2%; *P* = 0.001).

**Table 1:** Clinical characteristics of 463 patients with cancer of the esophagus or gastroesophageal junction who received neoadjuvant treatment

**Characteristic**	**Pathological complete responders**	**Pathological noncomplete responders**	***P*-value**
	***N*** **= 85 (18.4%)**	***N*** **= 378 (81.6%)**	
Median age in years (IQR)	63.8 (56.3–70.0)	63.1 (55.9–69.7)	0.729
Gender, *n* (%)			
Male	53 (62.4%)	293 (77.5%)	0.004
Female	32 (37.6%)	85 (22.5%)	
ASA score, *n* (%)			
ASA 1	20 (23.5%)	71 (18.8%)	0.526
ASA 2	50 (58.8%)	226 (59.8%)	
ASA 3	15 (17.6%)	81 (21.4%)	
Tumor histology, *n* (%)			
Adenocarcinoma	47 (55.3%)	270 (71.4%)	0.011
Squamous cell carcinoma	36 (42.4%)	105 (27.8%)	
Other	2 (2.4%)	3 (0.8%)	
Tumor location, *n* (%)			
Proximal esophagus	1 (1.2%)	0 (0.0%)	0.036
Mid-esophagus	22 (25.9%)	65 (17.2%)	
Distal esophagus	49 (57.6%)	234 (61.9%)	
Gastroesophageal junction	13 (15.3%)	79 (20.9%)	
Clinical T-stage, *n* (%)			
cT1	2 (2.4%)	5 (1.3%)	0.053
cT2	21 (24.7%)	51 (13.5%)	
cT3	58 (68.2%)	302 (79.9%)	
cT4	1 (1.2%)	2 (0.5%)	
cTx	3 (3.5%)	18 (4.8%)	
Clinical N-stage, *n* (%)			
cN0	17 (20.0%)	83 (22.0%)	0.076
cN1	34 (40.0%)	172 (45.5%)	
cN2	31 (36.5%)	93 (24.6%)	
cN3	0 (0.0%)	12 (3.2%)	
cNx	3 (3.5%)	18 (4.8%)	

*ASA* American Society of Anesthesiologist

**Table 2:** Treatment characteristics of 463 patients with cancer of the esophagus or gastroesophageal junction who received neoadjuvant treatment

**Characteristic**	**Pathological complete responders**	**Pathological noncomplete responders**
	***N*** **= 85 (18.4%)**	***N*** **= 378 (81.6%)**
Neoadjuvant treatment		
*Chemotherapy*	8 (9.4%)	80 (21.2%)
EOX	1	15
ECC	0	4
Cisplatin and etoposide	6	53
Cisplatin and etoposide	1	6
Hyperthermia		
Other chemotherapy scheme	0	2
*Chemoradiotherapy*	77 (90.6%)	298 (78.8%)
Radiotherapy & carboplatin and paclitaxel	65	238
Radiotherapy & carboplatin, paclitaxel and panitumumab	6	31
Radiotherapy & carboplatin, paclitaxel, and hyperthermia	4	327
Other chemoradiotherapy scheme	2	2
Surgical approach		
Transthoracic	72 (84.7%)	254 (67.2%)
Transhiatal	13 (15.3%)	124 (32.8%)

*ECC* epirubicin + cisplatin + capecitabine, *EOX* epirubicin + oxaliplatin + capecitabine

Table 3 describes the postoperative histopathology of both groups. Among the noncomplete responders, 11 patients (2.9%) had no vital tumor cells in the esophagus (tumor regression grade 1) but did have tumor-positive lymph nodes. Furthermore, in the resection specimen of 179 of the 378 patients with a noncomplete response (47.4%), no lymph node metastases (ypN0) was found, but vital tumor cells were still present in the esophageal wall. Groups did not differ in the median number of resected lymph nodes.

**Table 3:** Postoperative outcomes of 463 patients with cancer of the esophagus or gastroesophageal junction who received neoadjuvant treatment

**Characteristic**	**Pathological complete responders**	**Pathological noncomplete responders**	***P*-value**
	***N*** **= 85 (18.4%)**	***N*** **= 378 (81.6%)**	
Response (Mandard)			-
TRG 1	85 (100.0%)	11 (2.9%)	
TRG 2		67 (17.7%)	
TRG 3		132 (34.9%)	
TRG 4		57 (15.1%)	
TRG 5		37 (9.8%)	
Unknown/n.a		74 (19.6%)	
Radicality, *n* (%)			-
R0 (radical)	85 (100.0%)	355 (93.9%)	
R1 (microscopically irradical)		23 (6.1%)	
pT stage			-
pT0	85 (100.0%)	11 (2.9%)	
pT1		63 (16.7%)	
pT2		71 (18.8%)	
pT3		232 (61.4%)	
pT4		1 (0.3%)	
pN-stage			-
pN0	85 (100.0%)	179 (47.4%)	
pN1		108 (28.6%)	
pN2		64 (16.9%)	
pN3		27 (7.1%)	
Median number of LN harvested (IQR)	21 (15–27)	20 (15–28)	0.535
Median number of positive LN (IQR)	n/a	1 (0–2)	-

*IQR* interquartile range, *n.a.* not applicable, *TRG* tumor regression rate

### Survival Outcomes

**Fig. 1:**
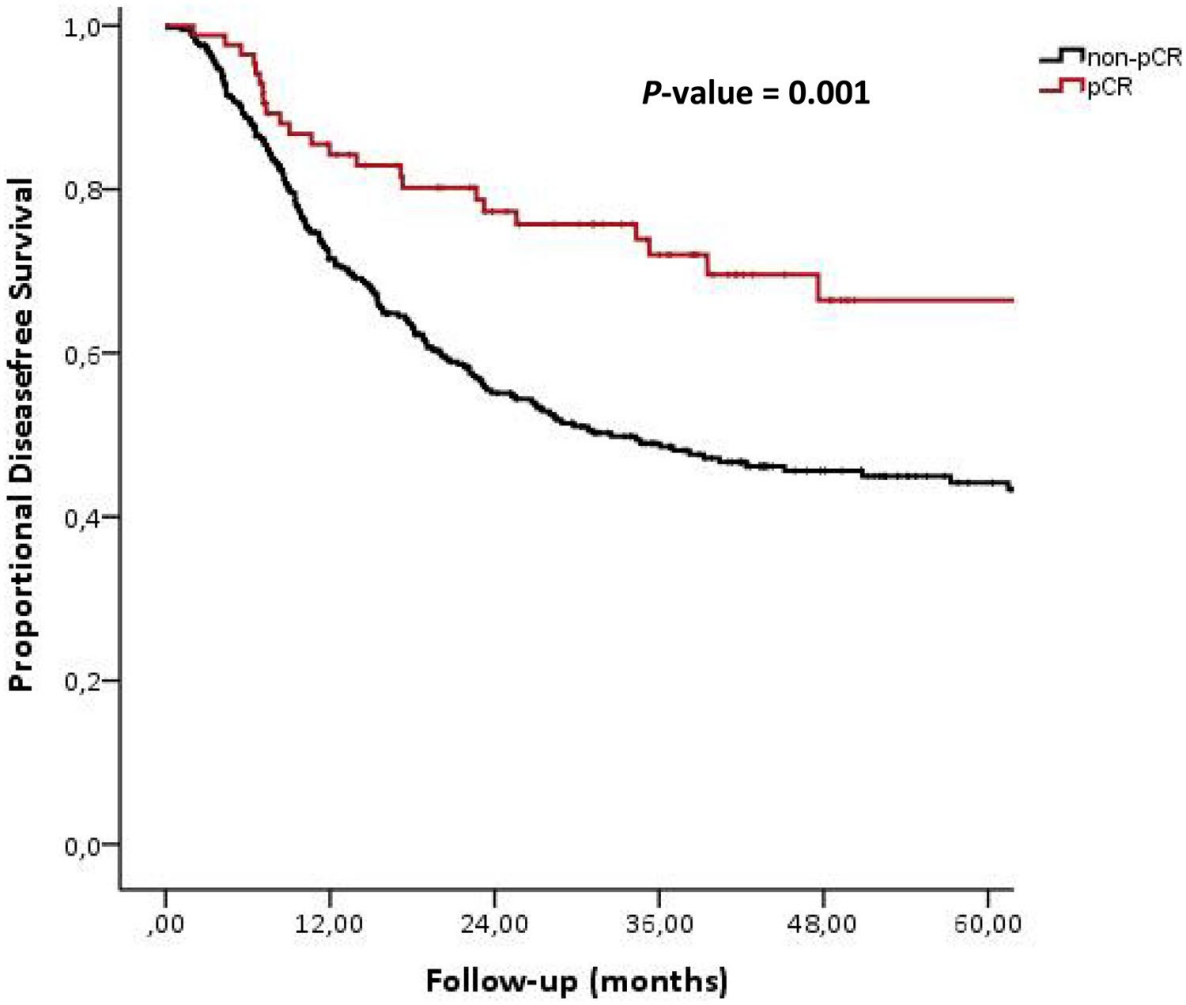
Disease-free survival of 463 patients with cancer of the esophagus or gastroesophageal junction who underwent neoadjuvant chemo(radio)therapy followed by surgery. Patients are divided between pathological complete responders (pCR, *n* = 85) vs. pathological noncomplete responders (non-pCR, *n* = 378)

**Fig. 2:**
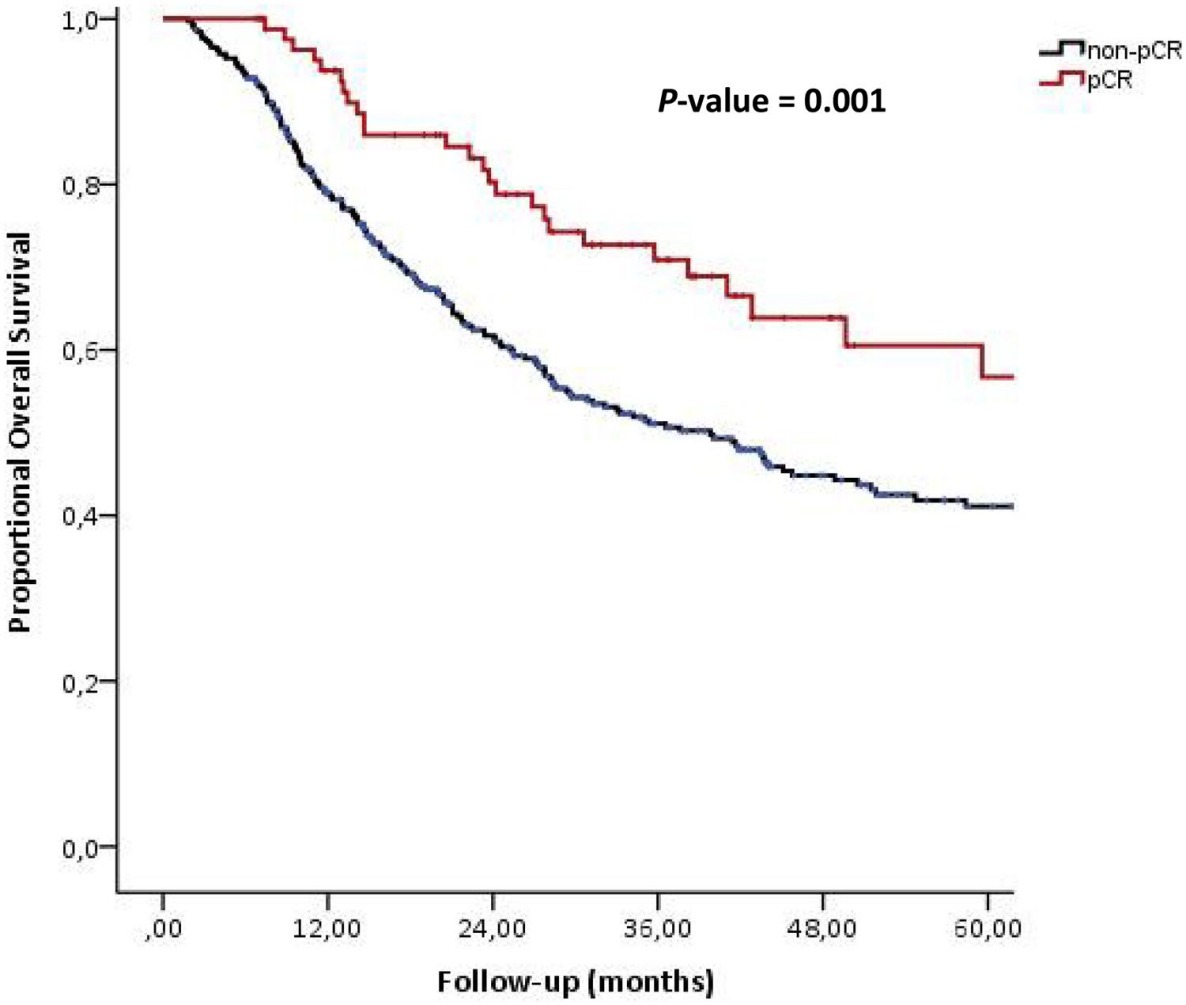
Overall survival of 463 patients with cancer of the esophagus or gastroesophageal junction who underwent neoadjuvant chemo(radio)therapy followed by surgery. Patients are divided between pathological complete responders (pCR, *n* = 85) vs. pathological noncomplete responders (non-pCR, *n* = 378)

A pCR was significantly associated with prolonged disease-free survival (*P* = 0.001) and overall survival (*P* = 0.001). Five-year disease-free survival rate in the pCR group was 69.6% and in the group of noncomplete responders 44.2% (Fig. 1). Five-year overall survival rates in the pCR group were 66.5% compared to 43.7% in the group of noncomplete responders (Fig. 2). The outcomes of a univariate analysis of both disease-free survival and overall survival are listed in Table 4. The factors that were statistically significant in predicting time to disease progression in the univariate model were ypT-stage, ypN-stage, and pCR. The same factors, supplemented with radicality, were significant predictors of overall survival in a univariate model. Table 5 shows the final multicovariate Cox model for disease-free survival and overall survival. Postoperative nodal stage (ypN-stage) is the only independent predictor for both disease-free survival and overall survival (*P* = 0.001).

**Table 4:** Univariate Cox model of prognostic factors in patients with cancer of the esophagus or gastroesophageal junction who underwent neoadjuvant chemo(radio)therapy followed by surgery

**Covariates**	**HR (95% CI)**	***P*-value**
***Disease-free survival***		
Age (> 65 years vs. < 65)	0.942 (0.715–1.242)	0.673
Gender (male vs. female)	1.147 (0.837–1.573)	0.394
Histology type	1.026 (0.815–1.291)	0.827
Tumor location	0.933 (0.753–1.156)	0.527
Neoadjuvant treatment (chemoradiotherapy vs. chemotherapy)	1.011 (0.724–1.412)	0.948
Operative approach (transthoracic vs. transhiatal)	1.115 (0.829–1.501)	0.471
ypT-stage	1.176 (1.078–1.283)	< 0.001
ypN-stage	1.719 (1.499–1.970)	< 0.001
Radicality	1.421 (0.793–2.546)	0.237
Complete pathological response (ypT0N0M0)	0.491 (0.323–0.746)	< 0.001
***Overall survival***		
Age (> 65 years vs. < 65)	1.162 (0.889–1.518)	0.271
Gender (male vs. female)	1.284 (0.936–1.762)	0.121
Histology type	0.949 (0.756–1.192)	0.655
Tumor location	0.998 (0.810–1.230)	0.988
Neoadjuvant treatment (chemoradiotherapy vs. chemotherapy)	0.919 (0.669–1.261)	0.599
Operative approach (transthoracic vs. transhiatal)	0.958 (0.723–1.270)	0.765
ypT-stage	1.200 (1.101–1.309)	< 0.001
ypN-stage	1.773 (1.551–2.027)	< 0.001
Radicality	1.813 (1.086–3.025)	0.023
Complete pathological response (ypT0N0M0)	0.528 (0.355–0.786)	0.002

*CI* confidence interval, *HR* hazard ratio

**Table 5:** Multicovariate Cox model of prognostic factors in patients with cancer of the esophagus or gastroesophageal junction who underwent neoadjuvant chemo(radio)therapy followed by surgery

**Covariates**	**HR (95% CI)**	***P*-value**
***Disease-free survival***		
ypT-stage	1.041 (0.920–1.178)	0.527
ypN-stage	1.641 (1.414–1.903)	< 0.001
Complete pathological response (ypT0N0M0)	0.832 (0.464–1.492)	0.537
***Overall survival***		
ypT-stage	1.101 (0.969–1.252)	0.139
ypN-stage	1.686 (1.456–1.951)	< 0.001
Radicality	1.319 (0.786–2.214)	0.295
Complete pathological response (ypT0N0M0)	1.119 (0.623–2.008)	0.707

*CI* confidence interval, *HR* hazard ratio

### Recurrence Pattern After pCR

During follow-up, disease recurrence was detected in 25 of the 85 patients (29.4%) with a pCR. In 19 (76%) patients, isolated distant metastases were detected. Three (12%) patients developed isolated locoregional recurrent disease, and in 3 (12%) patients, both distant and locoregional recurrences were found. Out of the 8 patients with a pCR after chemotherapy, 1 patient (12.5%) was diagnosed with recurrent disease in the form of isolated distant metastases. The remaining 24 cases (31.1%) of disease recurrence were found in the group of 77 patients with a pCR after chemoradiotherapy.

### FDG-PET/CT Performance in the Detection of pCR

A total of 135 (29.2%) patients underwent a restaging FDG-PET/CT after completion of neoadjuvant chemoradiotherapy. In 25 of these patients, a pCR was detected in the resection specimen. The diagnostic value of 2 SUV ratios ({SUVmax-tumor/SUVmax-esophagus} and {SUVmax-tumor/SUVmean-esophagus}) was assessed over a range of cut-off values (Table 6). There was no clear cut-off which would lead to a good discrimination between complete responders and noncomplete responders.

**Table 6:** FDG-PET/CT specifications in the detection of patients with a pathological complete response of the esophageal tumor on neoadjuvant chemoradiotherapy

**SUV**_**max-tumor**_**/SUV**_**max-esophagus**_				
**Cut-off value**	1.85	1.40	1.27	0.94
Specificity	0.40	0.28	0.24	0.08
Sensitivity	0.71	0.86	0.93	0.99
Positive predictive value	0.27	0.35	0.46	0.67
Negative predictive value	0.82	0.82	0.82	0.80

**SUV**_**max-tumor**_**/SUV**_**mean-esophagus**_				
**Cut-off value**	2.42	2.07	1.68	1.45
Specificity	0.40	0.32	0.16	0.12
Sensitivity	0.70	0.82	0.91	0.97
Positive predictive value	0.26	0.32	0.33	0.50
Negative predictive value	0.81	0.82	0.80	0.81

*SUV* standardized uptake value

Even a very low cut-off value with the highest sensitivity would only lead to a maximum positive predictive value of 67% for {SUVmax-tumor/SUVmax-esophagus} and 50% for {SUVmax-tumor/SUVmean-esophagus}.

## Discussion

The aim of this study was to determine the impact on (disease-free) survival of a pathological complete response after neoadjuvant therapy and surgical resection for esophageal cancer. In this cohort, a pCR rate of 18.4% (85/463) was observed which is comparable to literature [5]. Despite the fact that pCR was not identified as an independent predictor of survival, disease-free and overall survival were significantly longer in the group of patients with pCR compared to the group of noncomplete responders. Nevertheless, approximately one third of pCR patients developed recurrent disease, which consisted of distant metastases in more than 75% of cases. Furthermore, an analysis of 135 restaging FDG-PET/CT’s revealed no sufficient capacity to distinguish complete responders from noncomplete responders.

Our prognostic results are in accordance with recent reports on pCR in patient with esophageal cancer. In a study by van Hagen et al. [8], a pCR rate of 33.0% was shown after neoadjuvant chemoradiotherapy with a cisplatin- and 5-FU-based regimen. Within a follow-up time of 71.6 months, 39% of patients with a pCR developed recurrent disease. In only 6% of those patients, recurrent disease had an isolated locoregional character [8]. Lorenzen et al. recently described the outcomes of patients with a pCR after neoadjuvant docetaxel-/platinum-/fluoropyrimidine-based chemotherapy [9]. Eighteen out of 120 patients (15%) achieved a pCR, and this group was shown to have a significantly lower risk for tumor-related death compared with non-pCR patients (3-year cumulative incidences of 6.4% and 45.4%, respectively, P = 0.009). Currently, the largest study on this topic was performed by Vallböhmer et al. [12], who reported the outcomes of 299 patients with a pCR after either neoadjuvant radiochemotherapy (n = 284) or chemotherapy (n = 15). The disease-specific 5-year survival rate for this group of patients was 68%, with a recurrence rate of 23.4% (n = 70; local versus distant recurrence: 3.3% vs 20.1%).

The observed disease-specific 5-year survival rate in the present study (69.6%) is promising. However, a recurrence rate of 29.4% in patients with pCR is still high and implies that the systemic component of current neoadjuvant regimens, especially chemoradiotherapy regimens, is limited.

In the last few years, effort has been put in the development of models to detect the achievement of pCR in the preoperative phase [16, 23–26]. Until now, no diagnostic strategy has been formulated that successfully differentiates between complete and noncomplete responders. In previous studies on this topic, FDG-PET/CT has been the most frequently used diagnostic tool, but so far, the use of SUV ratios in the response assessment for esophageal cancer has never been described. By using SUV ratios, each patient is his or her own control because the surrounding (irradiated) esophagus is used as a reference [27]. Despite the limited number of analyzed FDG-PET/CT-CT scans and the absence of a baseline (pretreatment) FDG-PET/CT in this study, we feel that our results confirm previous reports on the restricted value of FDG-PET/CT in response evaluation [16]. Furthermore, based on their study on 138 patients, Heneghan et al. stated that even a combination of FDG-PET/CT and endoscopy is not reliable enough in terms of response evaluation [26]. The recently conducted pre-SANO trial [28] will have to prove whether complete response detection by a combination of endoscopy with (bite-on-bite) biopsies, EUS, fine-needle aspiration, and FDG-PET/CT is feasible and accurate. The ultimate goal of these studies is to adequately select a subgroup of patients with a “certain” pCR in order to prevent unnecessary surgery. However, one could argue that the currently observed recurrence rates after pCR do not (yet) give cause to such intentions.

We acknowledge the heterogeneity of our cohort with respect to tumor characteristics, neoadjuvant treatment, and surgical approach. The study is limited by its observational nature, especially since the described period is considerable. The observation that a larger part of the pCR patients underwent a transthoracic resection is explained by the fact that over time, parallel to improvement of neoadjuvant therapy, our center has been using the transthoracic approach increasingly in order to guarantee optimal lymphadenectomy [21, 29]. Additionally, squamous cell carcinomas, known for a better response to chemoradiotherapy compared to adenocarcinomas [6], were more likely to be treated with a transthoracic resection because of their common location proximal to the gastroesophageal junction.

In conclusion, the observations in this representative cohort confirm the important finding that detection of a pCR does not mean that the ultimate goal of cure has been achieved since patients with a pCR still bear a considerable risk of recurrent disease. Furthermore, the results of this study show that FDG-PET/CT cannot be used as a sole diagnostic tool to predict pCR after CRT for esophageal cancer. In general, one could state that next to adequate response prediction, an equally relevant scientific challenge is to be found in the improvement of the systemic impact of (neo)adjuvant treatment.

## Data Availability

Data are available at the corresponding author upon reasonable request.
